# Combined Near-Infrarred Light Transillumination and Direct Digital Radiography Increases Diagnostic In Approximal Caries

**DOI:** 10.1038/s41598-019-50850-5

**Published:** 2019-10-02

**Authors:** Maria Melo, Agustin Pascual, Isabel Camps, Fadi Ata-Ali, Javier Ata-Ali

**Affiliations:** 1 Valencia University Medical and Dental School, University of Valencia, Valencia, Spain; 2 Universidad Europea de Valencia. Faculty of Health Sciences. Department of Dentistry, Valencia, Spain; 3 Public Dental Health Service. Conselleria de Sanitat Universal i Salut Pública, Generalitat Valenciana, Valencia, Spain

**Keywords:** Dental diseases, Oral diseases

## Abstract

The objective of this study was to evaluate the clinical ability of Near-Infrared Light-Transillumination (NILT) for approximal dentinal caries detection and to compare with direct digital-radiography (DDR), as well as to determine whether the combination of both techniques improves the diagnostic capacity of the lesions. From 88 patients (over 18 years), 138 posterior teeth (76 molars and 62 premolars), that had approximal caries reached into dentine determined by DDR, were included. Lesion extension and DDR images were scored as follows: D0 = sound surface, D1/D2 = caries restricted to the outer/inner-half of the enamel, and D3/4 = caries restricted to the outer/inner-half of the dentin. Opening of the approximal surface using 0.5 mm-in-diameter diamond-bar was used as gold-standard. The lesion extension was then determined by the following criteria: no dentinal caries (D0/1/2) or dentinal caries (D3/4). Seventy-one lesions were D3 and 67 lesions were D4. Sensitivities of overall/D3/D4 were 98.0/95.7/100.0 (NILT) and 100/100/100 (DDR), respectively. Correlations with gold-standard were 0.92 (NILT) and 0.42 (DDR), respectively. The correlation increased to 0.97 (p = 0.045) on combining NILT and DDR. There was no difference in sensitivity between the methods (p > 0.05); but was differences in the correlation (p < 0.01). It can be concluded that NILT showed sensitivity similar to that of DDR and higher correlation than DDR for approximal dentinal caries detection. Accordingly, it may be used to monitor the progression of caries without exposing the patient to ionizing radiation, this being of particular interest in growing patients and in pregnant women. In this respect, NILT can be an effective diagnostic tool adjunctive to bitewing radiographs in detecting approximal dentinal caries. The combination of NILT and DDR represents an increase in the diagnosis of approximal lesions The proposed diagnostic protocol comprises visual examination, followed by NILT and DDR only if the former technique detects approximal caries.

## Introduction

The detection of early stage caries has become an objective for clinicians and investigators, because it allows the implementation of minimally invasive therapies. Early detection has multiple advantages, and noninvasive treatment strategies such as the remineralization of early stage of caries lesions, infiltration or ozonization, among other options, are of interest in preserving as much dental tissue as possible^1^. A number of studies have evaluated the effects of mineralization with fluoride or fluoride combined with bioadhesive polymers, or casein phosphopeptide-amorphous calcium phosphate with favorable results^2–4^. Resin infiltration is another valid option^5–8^ when the lesions are not cavitated and are radiographically limited to the enamel or external third of the dentin layer^9^. On the other hand, some authors consider that this type of treatment requires further research^10^. The microinvasive treatment of approximal lesions significantly reduces their risk of progression when compared with noninvasive treatments such as plaque control^6^.

The traditional clinical methods (visual and tactile examinations) are considered to be insufficient for detecting approximal caries^11^. The International Caries Detection and Assessment System (ICDAS) was developed in 2002 and introduces systematic scoring definitions for better classification of caries at different stages. This 6-score ordinal system registers caries from the initial changes in the enamel to extensive cavities in dentin^12^. Bitewing radiographs have represented the gold standard for detecting approximal caries^13^, but the indication and repeatability of dental X-rays are considerably limited, because of the associated ionizing radiation. In this regard, the development of alternative detection methods affording high sensitivity and specificity without the use of ionizing radiation is important in order to allow more conservative dental practice.

Different methods based on optical principles have been developed with the aim of detecting the initial enamel changes in caries. Near Infra-red Light Transillumination (NILT) is a photo-optical method for caries detection and diagnosis in posterior teeth, and was first described in 1995^14^. It is a development of the digital imaging fiber-optic transillumination method (DIFOTI) that uses visible light, and which in turn is an evolution of the FOTI (fiber-optic transillumination) method. The NILT based method used in our study operates at a wavelength of 780 nm, and was introduced on the European dental market in 2012^15^. Transillumination of tooth surfaces with light at specific wavelengths has shown good potential for the detection of incipient lesions, because it allows for differentiation between healthy and carious tissue^15,16^. Because tooth enamel is highly transparent in this region, NILT presents low scattering and absorption at these wavelengths^16^. Laboratory study presented that NILT demonstrated a potential for early approximal caries detection^17^. It is advisable to conduct *in vivo* studies with a large sample size in order to determine whether the detection capacity of NILT is effectively comparable to that of radiography. If this indeed proves to be the case, then approximal caries could be diagnosed and monitored without exposure to ionizing radiation – this being a clear advantage for patients. In contrast to other methods, NILT has been shown to differentiate demineralization from other changes in enamel such as pigmentation, fluorosis, calculus, fissures or developmental defects^17^. Another important modification with respect to the classical transillumination technique is that in the case of NILT the light is shone around the alveolar process, not directly in the approximal space^15^. Because of its recent introduction, few clinical studies using this technology can be found in the literature. Thus, is considered necessary to perform a greater number of clinical studies with validation of the extent of the lesion after opening the cavity in order to determine (or not) in the future the use of this system as a gold standard for the detection of proximal caries. It is also necessary to establish whether combination of the different methods is able to increase the percentage of cases correctly diagnosed as approximal caries.

The literature contains both *in vitro*^17^ and *in vivo* studies^18,19^, though they are limited in number. Many studies have focused on the diagnosis of occlusal caries^1,11,20–22^, though few authors have addressed approximal caries^13,15,19,23,24^. In this respect, the present report describes one of the largest series of patients in an *in vivo* prospective study assessing an NILT method for the detection of approximal dentinal caries, establishing comparisons with digital bitewing radiographs, and where the true lesion depth is assessed by opening the cavity. In addition, we present a diagnostic strategy to be followed in our patients and examine whether the combination of both proposed methods is able to improve the capacity to detect carious lesions. The working hypothesis is that by knowing the extent of a lesion with DDR it is possible to predict the extent of that same lesion with NILT. As null hypothesis, we postulated that NILT does not exhibit significant differences with respect to intraoral radiographs in detecting approximal caries with dentinal involvement.

## Subjects and Methods

### Patients

A prospective study was made of 112 patients in the Department of Dentistry (University of Valencia, Spain) between 1 February 2016 and 31 December 2016. Informed consent was obtained from all participants in this study. The study and the experimental protocol were reviewed and approved by the Institutional Review Board of the University of Valencia (IRB H1446929644201), and has been conducted in accordance with the Declaration of Helsinki.

All patients were classified as ASA I (healthy individuals) or ASA II (patients with mild, controlled and non-disabling disease), and all were at least 18 years old. All the patients were seen for a routine dental visit and were evaluated with visual examination by ICDAS, near-infrared light transillumination (NILT) and the direct digital radiography (DDR). All permanent premolars and molars were evaluated. The following teeth were excluded: teeth with previous restorations or with sealing of pits; extensive caries and visible dentin on free and approximal smooth surfaces; enamel anomalies and dentinal alterations such as fluorosis, hypoplasia or dentinogensis/amelogenesis imperfecta. The analytical unit was the tooth, not the approximal surface, since in the case of teeth with caries affecting both approximal surfaces, we recorded that with the greatest disease involvement. It was no indispensable the existence of a contact point.

A total of 1283 teeth were analyzed. Sound surface was 1806/1594 (DDR/NILT), enamel caries was 261/348, and dentinal caries was 138/162, respectively (Table 1). Accordingly, we only included the dentin lesions detected by the DDR. A total of 138 teeth (76 molars and 62 premolars) from 88 patients (49 females and 39 males) with a mean age of 42.3 ± 12.4 years were included in this study.

**Table 1:** Prevalence of D0, D1 and D2 lesions according to ICDAS, DDR and NILT. ICDAS: International Caries Detection and Assessment System.

	ICDAS	DDR	NILT
D0	3400	1806	1594
D1	0	82	179
D2	0	128	220

DDR: Direct digital radiography.

NILT: Near infrared light transillumination.

### Near infrared light transillumination (NILT)

All the premolars and molars included in the study were examined using the NILT system (DIAGNOcam, Kavo, Biberach, Germany). After air syringe drying the included approximal sites, the sites were examined over and perpendicular to the region of interest. The images were captured always by the same operator (MM) and stored with KID software (Kavo Integrated Desktop, Kavo, Biberach, Germany). Later stored NILT images were evaluated by two examiners (M.M and A.P). A consensus NILT score was determined by the following criteria: sound surface, dark-shadow in the outer-half of the enamel, dark-shadow in the inner-half of the enamel, dark-shadow past dentin-enamel junction (DEJ) and lightly involved dentin, and dark-shadow past DEJ and heavily involved dentin. At least, two images of each tooth were taken in order to obtain a good view.

### Direct digital radiography (DDR)

All intraoral DDR images was acquired with the same radiological device (Gendex Oralix, Gendex, Milan, Italy) the same day as NILT examination. The bitewing radiographs were taken as a standarized protocol of review by one examiner (M.M). Later, all saved DDR images were analyzed by two examiners (M.M and A.P). All the images were evaluated independently from all the other diagnostic findings using the available software (Sirona, Bensheim, Germany), with the option of enhancing brightness and contrast for better image assessment. For each surface, the corresponding categorical diagnosis (D-score) was made as follows: D0 = sound surface; D1 = caries restricted to the outer half of the enamel, D2 = caries restricted to the inner half of the enamel; and D3/4 = caries restricted to the outer/inner half of the dentin^25^. In this regard, the D1 and D2 lesions were both limited to the enamel, though the difference between them refers to the extent of the lesions: if enamel thickness is divided in two, D1 would correspond to lesions located between the most external portion and the indicated half thickness of the enamel layer, while D2 would surpass this half way point and could even reach the amelodentinal junction. All radiographic decisions were re-assessed before the next patient visit, in order to establish a consensus-based diagnosis. When different results were obtained by the two examiners, the radiographs were re-evaluated, commented, and a consensus-based conclusion was drawn. Independent analyses of all the images obtained DDR were carried out on a blind basis with a minimum interval of two weeks after NILT examination.

### Extent of the carious lesions (reference gold standard)

Due to ethical reasons, we only validated the diagnosis in the case of those teeth yielding a score of D3 or D4 from the DDR. The enamel lesions detected in the study are susceptible to be treated with non-invasive methods such as remineralization or infiltration with resins^26,27^. For this reason, D1 and D2 lesions were not validated by opening the cavity because it was an *in vivo* study. The frequency of revisions and bitewing radiographs will depend on the level of risk of each patient, and it must be an individualized decision.

The validation was done between 3 and 7 days after taking the images. The extent of the lesion was then determined according to the following scale: no dentinal caries (D 0 + 1 + 2) or dentinal caries (D 3 + 4)^28^. The true extent of the carious lesions was first determined using round diamond drills measuring 0.5 mm in diameter (Komet, Gebr. Brasseler GmbH & Co., Lemgo, Germany). The final lesion depth reached was used for the study, and was corroborated visually and with the tip of the exploratory probe, assessing the hardness of the bottom of the cavity (M.M). This was followed by filling of the cavity in the same operation.

### Statistical analysis

The data were analyzed using the SPSS version 20.0 statistical package (IBM Corp., Armonk, NY, USA). We calculated the sensitivity, positive predictive value, true-positive and -negative results, and false-positive and -negative results of each diagnostic method. The linearly weighted Kappa index was estimated to assess the agreement in diagnostic accuracy of the different methods (D1, D2, etc.) and the true extent of the lesion (gold standard). Theoretical sensitivity values of 90% and 96% for two diagnostic methods (OR = 2.5) were established as statistically significant using a related samples proportions test (such as the McNemar test) with a statistical power of 80.5% for a 95% confidence level. The required sample size was previously calculated using the G*Power application (University of Düsseldorf). The reproducibility intra and inter-examiner was calculated with Cohen’s Kappa coefficient. Simple and multiple logistic regression models were used to establish the diagnostic correlation indicators of the combined methods.

## Results

A total of 138 teeth (76 molars and 62 premolars) from 88 patients (49 females and 39 males) with a mean age of 42.3 ± 12.4 years were included in this study. Of the 138 teeth, 71 (51.4%) and 67 (48.6%) were D3 and D4 lesions, respectively.

Table 2 shows the sensitivity and correlation with gold standard (reference method). For sensitivities in any levels between NILT and DDR, there was no significant difference (p > 0.05). For correlations between NILT and DDR, there was significant difference (p < 0.01). The combination of NILT and DDR significantly improves correlation with the true extent of the lesion (0.97; p = 0.045).

**Table 2:** Overall sensitivity and based on lesion extension, and correlation with histology. Superscript letters indicate there are no significant differences between the methods in sensitivity, but there are differences in the correlation.

Method	Sensitivity			Correlation
	Overall (n = 138)	D3 (n = 71)	D4 (n = 67)	
NILT	98.0^a^	95.7^a^	100.0^a^	0.92^a^
DDR	100.0^a^	100.0^a^	100.0^a^	0.42^b^
ICDAS	38.4^a^	26.8^a^	50.7^a^	0.24^b^

NILT: Near infrared light transillumination.

DDR: Direct digital radiography.

^a^p-value < 0.05.

^b^p-value > 0.001.

The reproducibility inter and intra examiner is higher for NILT tan DDR. For the first method, the interexaminer reproducibility is 0.83 while DDR obtains 0.75. The intraexaminer reproducibility is also greater for NILT (0.88–0.92) tan DDR (0.78–0.8) (Table 3).

**Table 3:** Reproducibility of examiners.

	Examiner 1	Examiner 2	Interexaminer
ICDAS	0.63 (0.54–0.80)	0.77 (0.57–0.90)	0.58 (0.33–0.76)
DDR	0.78 (0.62–0.83)	0.80 (0.55–0.88)	0.75 (0.69–0.83)
NILT	0.88 (0.67–0.92)	0.92 (0.74–0.94)	0.83 (0.71–0.89)

**Figure 1:**
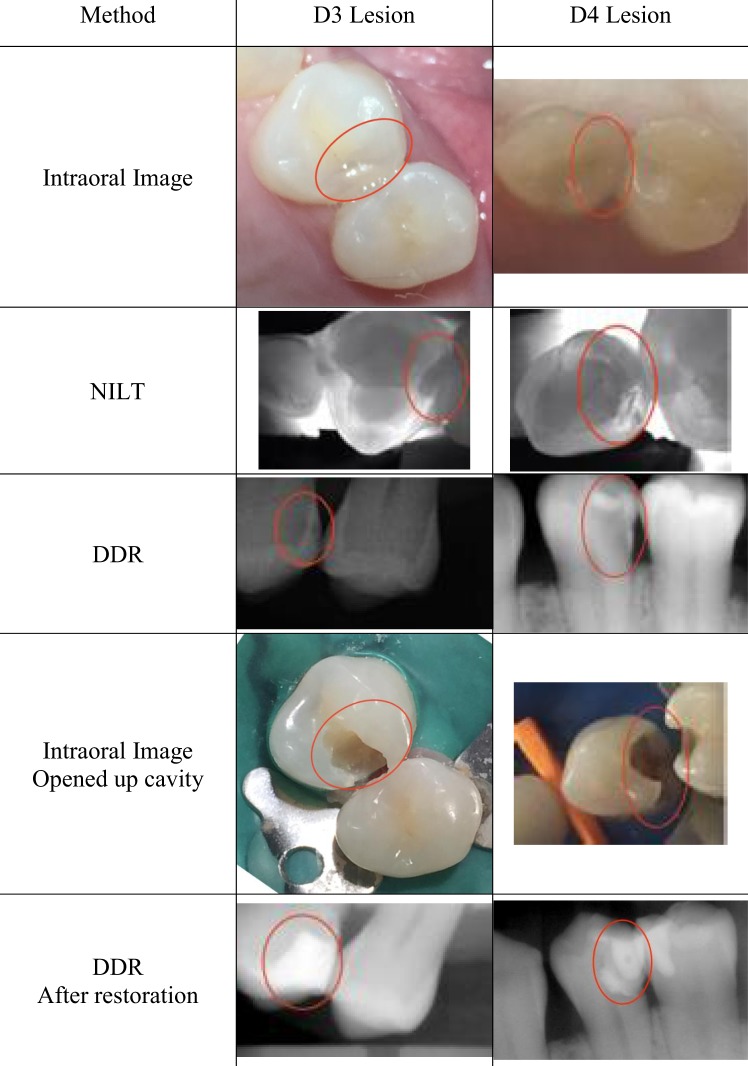
Examples of D3 and D4 lesions for each method: Intraoral image, NILT: Near infrared light transillumination, and DDR: Direct digital radiography. Orange circles indicate the location of lesion. D3 lesion was confirmed on the distal surface of the maxillary left first premolar. D4 lesion was confirmed on the distal surface of the mandibular left first premolar.

Figure 1 shows example images of D3 and D4 lesions. For D3 lesion, the NILT image showed dark-shadow past DEJ and lightly involved dentin. After opened up cavity, dentinal caries was clearly indicated. For D4 lesion, the NILT image showed dark-shadow past DEJ and heavily involved dentin. After opened up cavity, dentinal caries was clearly showed.

## Discussion

This study evaluated the null hypothesis that the near-infrared light transillumination (NILT) method does not show significant differences versus intraoral radiographs in detecting approximal dentinal caries. It seems supported by the results. An early detection offers the possibility of using noninvasive treatments such as resin infiltration or ozone therapy, resulting in greater dental tissue preservation^4,7,8,29^. Different techniques have been developed to aid in caries detection, which is the starting point for establishing suitable treatment^1^. For this current study, due to ethical reasons, we didn’t validate the caries detected in enamel with DDR or NILT. For this reason, we were unable to calculate specificity, negative predictive value and the area under the receiver operating characteristic curve. Other studies that included negative sample were either *in vivo* studies in which validation was justified by the fact that the examined teeth were to be used as abutments for fixed prostheses^30,31^, or *in vitro* studies^1,32–35^. One of the advantages of this current clinical study is that the real extension of the lesion is obtained opening up the cavity.

The present study was designed to evaluate this NILT method for detection of approximal dentinal caries, comparing it with bitewing direct digital radiography (DDR). The results indicated that the NILT method showed overall high sensitivity (98%), and higher than those reported in other studies^15,18^. Even in *in vitro* studies the sensitivity obtained with NILT is lower. The study published by Abogazalah *et al*.^17^ recorded a sensitivity of 68% for NILT, similar to that obtained by Elhennawy *et al*.^36^ (63% in teeth with restorations in proximal cavities). Baltacioglu *et al*.^23^ recorded an area under the ROC curve of between 0.785 and 0.832, but did not specify the absolute sensitivity and specificity values, and the study sample size was moreover small.

The study published by Lara-Capi^37^ compared NILT and radiographic examinations at dentinal level, but this study did not include any gold standard reference method. Both methods identified the same number of carious lesions (kappa = 1), whereas more approximal lesions were recorded using the NILT in enamel (kappa = 0.24). Although our current study did not include enamel caries lesions, these findings are consistent with those of our own study, where enamel lesions were observed in 261 teeth with the radiographic technique and in 348 teeth with NILT. Retrospective analysis showed that agreement between NILT and bitewing radiography was similar for dental caries detection^19^. Our current study is in agreement with these other studies. In terms of repeatability, other study indicated good to excellent inter- and intra-examiner agreement using NILT for approximal caries detection^18^. The results from this current study showed that NILT presented significantly higher correlation with gold standard than DDR, in the same way as in the study of Abogazalah *et al*.^17^ though it must be noted that this study was conducted *in vitro*. This may be because tooth enamel is highly transparent, and NILT presents low scattering and absorption at wavelength of 780 nm from NILT^16^. DDR can detect a demineralization when the mineral loss is 30%, but no before it normally^38^. Transillumination of tooth surfaces with light at specific wavelengths has shown good potential for the detection of early stage of caries lesions, because it allows for differentiation between healthy and carious tissue^1,15,16^. Based on these findings for NILT and previous study^17^, we developed and employed the NILT score system in this current study. Since the correlation with gold standard showed moderate, this NILT score seems effective, especially for dentinal caries detection and quantification. Further study will need to confirm and establish enamel caries detection/quantification.

We have observed cases in which caries extended to the dentin on the radiographs, but without apparent cavitation. In our current study, the radiographic method was found to be the most sensitive, since no detection failures were recorded. One of the disadvantages of the radiographic method is exposure to ionizing radiation^39,40^. Accordingly, its use should be justified on an individual basis, avoiding its indiscriminate application^41^. The ALARA (as low as reasonably achievable) principle holds that the desired amount of information must be obtained with the smallest possible amount of radiation^38,42^. At a lower dose, noise will increase and the diagnostic value of the radiograph will decrease. It is true that all digital devices require less radiation than film for obtaining diagnostically “just acceptable” radiographs, although the amount of reduction differs significantly^42,43^.

Caries lesions classified as D1 and D2 cannot be validated by opening the cavity because doing so would represent overtreatment^26^. This principle is currently becoming consolidated, and establishes the bases of clinical practice seeking to adopt a noninvasive approach in dealing with these lesions. Accordingly, as indicated by Ozkan and Guzel^24^, a lesion that does not reach the amelodentinal junction should not be opened as per preventive dentistry norms^44,45^. The sensitivity values reported by Ozkan were 0.54, 0.83 and 0.82 for the visual, DDR and NILT methods, respectively^24^. These figures are slightly lower than our own (0.38, 1 and 0.98), possibly because in our study the inclusion criteria comprised caries with a DDR score of D3 or D4. However, the specificity values (0.60–0.20) and negative predictive values (0.10–0.03) showed greater differences in favor of the intraoral radiographs. In coincidence with other studies, visual assessment was not appropriate for diagnosing approximal caries^24,40,44^.

As clinical protocol, we recommend the ICDAS visual method to be used first (in the awareness that this only allows us to detect 38.4% of the lesions), followed by NILT (reaching a sensitivity of 98%, with p < 0.05 versus DDR). Only if NILT effectively diagnoses approximal caries do we then apply DDR, thereby significantly reducing patient exposure to ionizing radiation – this being of particular interest in growing patients and in pregnant women. For example, in cancer diagnosis, screening has focused on high-risk populations^46^; as a result, patients not belonging to this high-risk group would not be diagnosed. However, sensitivity and specificity are not the only factors to be taken into account in diagnostic reasoning^47^. In our case, the fact that NILT is used first eliminates exposure to ionizing radiation, even if the dose involved is very low – since the effect of radiation exposure is cumulative.

It is important to point out that none of the methods analyzed in this study are able to diagnose 100% of all the carious lesions. In contrast to what might be believed, the strength of DDR is precisely the main limitation of NILT: while DDR affords very precise visualization of the pulp chamber, in the case of NILT carious lesions at this level cannot be reliably diagnosed. Deep interproximal lesions (D4) that are not limited to the outer half of the dentin can be seen as shadows of lesser translucency within the dentin with the NILT system, and interpretation of the images may prove difficult. Although not the objective of this study, it should be noted that NILT also offers the possibility of introducing a third dimension in the diagnosis of carious lesions, and that this aspect is crucial in those cases where conventional treatment (opening of the cavity) is required – since it allows us to know the precise buccolingual location of the lesion and thus act more conservatively in dealing with it.

Within the limitations of this study, it can be concluded that NILT showed sensitivity similar to that of DDR and higher correlation than DDR for approximal dentinal caries detection. This indicates that correlation with the true extent of the lesion is better with NILT, with DDR tending to underestimate the extent of approximal carious lesions. Accordingly, NILT may be used to monitor the progression of caries without exposing the patient to ionizing radiation – this being particularly interesting in growing patients and in pregnant women. In this respect, NILT can be an effective diagnostic tool adjunctive to bitewing radiographs in detecting approximal dentinal caries. The combination of NILT and DDR represents an increase in the diagnosis of lesions. The proposed diagnostic protocol comprises visual examination, followed by NILT and DDR only if the former technique detects approximal caries.
